# Impact of the COVID‐19 pandemic on breast, colorectal, lung, and prostate cancer stage at diagnosis according to race

**DOI:** 10.1002/cam4.5439

**Published:** 2022-11-20

**Authors:** Jennifer Berrian, Ying Liu, Nkiruka Ezenwajiaku, Alvaro Moreno‐Aspitia, Sara J. Holton, Adetunji T. Toriola, Graham A. Colditz, Ashley J. Housten, Lannis Hall, Mark A. Fiala, Foluso O. Ademuyiwa

**Affiliations:** ^1^ Washington University School of Medicine St. Louis Missouri USA; ^2^ University of Miami Coral Gables Florida USA; ^3^ Mayo Clinic Jacksonville Florida USA

## Abstract

**Purpose:**

To determine if the COVID‐19 pandemic has further exacerbated racial disparities in late‐stage presentation of breast, colorectal, lung, and prostate cancers.

**Methods:**

We conducted a registry‐based retrospective study of patients with newly reported diagnoses of breast, colorectal, lung, and prostate cancers between March 2019–June 2019 (pre‐COVID‐19) and March 2020–June 2020 (early‐COVID‐19). We compared the volume of new diagnoses and stage at presentation according to race between both periods.

**Results:**

During the study period, a total of 3528 patients had newly diagnosed cancer; 3304 of which had known disease stages and were included in the formal analyses. 467 (14.1%) were Blacks, and 2743 were (83%) Whites. 1216 (36.8%) had breast, 415 (12.6%) had colorectal, 827 (25%) had lung, and 846 (25.6%) had prostate cancers, respectively. The pre‐COVID‐19 period included 2120 (64.2%), and the early‐COVID‐19 period included 1184 (35.8%), representing a proportional 44.2% decline in the volume of new cases of breast, colorectal, lung, and prostate cancers, *p* < 0.0001. Pre‐COVID‐19, 16.8% were diagnosed with metastatic disease, versus 20.4% early‐COVID‐19, representing a proportional increase of 21.4% in the numbers of new cases with metastatic disease, *p* = 0.01. There was a non‐significant proportional decline of 1.9% in Black patients diagnosed with non‐metastatic breast, colorectal, lung, and prostate cancers early‐COVID‐19 (*p* = 0.71) and a non‐significant proportional increase of 7% in Black patients diagnosed with metastatic disease (*p* = 0.71). Difference‐in‐difference analyses showed no statistically significant differences in metastatic presentation comparing Black to White patients.

**Conclusion:**

While we identified substantial reductions in the volume of new cancer diagnoses and increases in metastatic presentations due to the COVID‐19 pandemic, the impact was similar for White and Black patients.

## Graphical abstract

Following the declaration of COVID‐19 as a global pandemic, there was a significant decline in the volume of newly diagnosed cases of breast, colorectal, lung, and prostate cancers. We observed an increase in the proportion of patients diagnosed with metastatic disease. The impact was similar for White and Black patients as there was no significant racial difference in the proportion of cases presenting with metastatic disease.

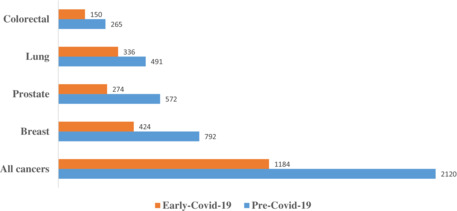

## 1 INTRODUCTION

While screening guidelines and public education campaigns have decreased cancer mortality rates,^1^ in Black patients with cancer, disparities still exist when compared to White patients.^2^, ^3^ In particular, when looking at breast, colorectal, lung and prostate cancer, Black patients experience higher rates of morbidity and mortality.^4^, ^5^, ^6^ The factors responsible for these disparities are myriad and include socioeconomic factors,^7^, ^8^ insurance status,^9^, ^10^ access to screening and treatment facilities,^11^ unconscious biases and unbalanced patient‐provider relationship dynamics,^12^, ^13^ and more.

Independent of other factors, being diagnosed at a later stage worsens prognosis and increases the likelihood of cancer‐related mortality. When exploring mortality rates between Black and White cancer patients, research shows an increase in incidence rates of advanced stage cancers in Black patients.^14^ This trend holds true across the four major cancers included in our study; breast,^15^ prostate,^16^ lung,^17^, ^18^ and colorectal.^19^ This trend in increased incidence of advanced stage cancers in Black patients must be considered and addressed when exploring disparities in cancer‐specific mortality between Black and White cancer patients.

The COVID‐19 pandemic has resulted in disruptions in cancer care including overdue cancer screening,^20^, ^21^ delays in diagnosis,^22^ decreases in resources and treatment delay,^23^, ^24^ and increases in cancer morbidity and mortality rates.^25^, ^26^ Coupled with the historically higher rates of more advanced stage at presentation in Black patients, we hypothesized that the COVID‐19 pandemic has further exacerbated racial disparities in stage at presentation for patients with newly diagnosed cancers.

## 2 METHODS

### 2.1 Patient selection

Data from the Barnes Jewish Hospital Cancer Registry, the University of Miami Health Center Sylvester Cancer Center Tumor Registry, and the Mayo Clinic Tumor Registry were used for this study. The Washington University School of Medicine Institutional Review Board (IRB ID #202103105) approved this study. Informed consent was waived because the data were anonymous, and the study posed minimal risk to participants, in accordance with 45 CFR §46. For this multi‐institutional registry‐based cross‐sectional study, we included all newly diagnosed adult cases of breast, colorectal, prostate, and lung cancer submitted to all three registries. In order to evaluate the impact of the COVID‐19 pandemic on the numbers of new diagnoses and stages of diagnoses of these four cancers, we obtained data from cancer cases diagnosed in the periods between March 2019–June 2019 (pre‐COVID‐19) and March 2020–June 2020 (early‐COVID‐19). This pre‐COVID‐19 timeframe was selected as a control timeframe with similar months during the year immediately prior to the COVID‐19 pandemic. The early‐COVID‐19 timeframe was selected as this was when the most disruptions occurred in healthcare. Cases with unknown staging or race were excluded. We retrieved detailed data on primary cancer (breast, colorectal, prostate, or lung), race (White, Black, Other), date of diagnosis, age at diagnosis, stage at diagnosis, sex, and insurance type (Medicare, Medicaid, private, other, and uninsured). Disease stage was defined as per the 8th edition of the American Joint Committee on Cancer (AJCC) tumor, node and metastasis (TNM) classification. Reporting of results follows the Strengthening the Reporting of Observational Studies in Epidemiology (STROBE) reporting guideline.^27^

### 2.2 Statistical analyses

We calculated the percentages of metastatic diagnoses pre‐COVID‐19 and during early‐COVID‐19 by race, health insurance, and types of cancer. We used logistic regression to estimate the odds ratio (OR) of being diagnosed with metastatic cancer in early‐COVID‐19 pandemic compared to pre‐COVID‐19 pandemic. The OR was adjusted for age, race, health insurance, types of cancer, and study cohorts. Difference‐in‐differences analysis was performed to test the significance of COVID‐19 associated percent changes in metastatic diagnoses, using linear probability models adjusted for age, race, types of cancer, and study cohorts. All statistical analyses were performed using SAS (version 9.4; SAS Institute, Cary, NC). Statistical significance was assessed as two‐sided *p* < 0.05.

## 3 RESULTS

### 3.1 Patient characteristics

During the study period, 3528 individuals were newly diagnosed with either breast, colorectal, lung, or prostate cancers. 3304 had known disease stage reported and were included in the formal analyses. Overall, 467 (14.1%) were Black, 2743 (83%) were White, and 94 (2.9%) were other races. The study included 1216 (36.8%) with breast, 415 (12.6%) with colorectal, 827 (25.6%) with lung, and 846 (25.6%) with prostate cancer respectively (Table 1). Women comprised 54.4% of the entire cohort, and the median (IQR) age was 64 (56–72) years. Insurance type was designated as Medicaid for 136 (4.1%), Medicare for 1537 (46.5%), private for 1446 (43.8%), other for 155 (4.7%), and uninsured for 30 (0.9%).

**TABLE 1:** Characteristics of the study population

Pre‐Covid‐19		Early‐Covid‐19
*N* = 2120 (%)		*N* = 1184 (%)
Disease site		
Breast	792 (37.4)	424 (35.8)
Colorectal	265(12.5)	150 (12.7)
Lung	491 (23.2)	336 (28.4)
Prostate	572 (26.9)	274 (23.1)
Age (year, median, IQR)	65 (57.0–72.0)	64 (56.0–71.5)
Female, *N* (%)	1222 (54.0%)	697 (55.1)
Race		
White	1747 (82.4)	996 (84.1)
Black	300 (14.2)	167 (14.1)
Other race	73 (3.4)	21 (1.8)
Insurance type		
Medicaid	87 (4.1)	49 (4.1)
Medicare	993 (46.8)	544 (45.9)
Private	918 (43.3)	528 (44.6)
Other	101 (4.8)	54 (4.6)
Uninsured	21 (1.0)	9 (0.8)

Abbreviations: IQR, interquartile range; *N*, number.

### 3.2 Impact of the COVID‐19 pandemic on volume of new diagnoses

**FIGURE 1:**
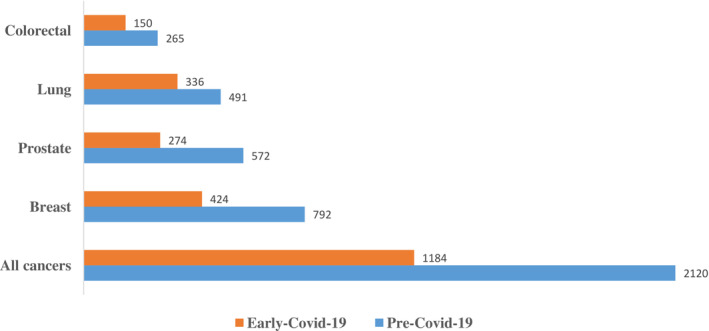
Impact of the COVID‐19 pandemic on the absolute numbers of new diagnoses of breast, colorectal, lung, and prostate cancers. Absolute numbers of new reported diagnoses of all breast, colorectal, lung, and prostate cancer pre‐COVID‐19 and early‐COVID‐19. Blue bars represent pre‐COVID‐19; red bars represent early‐COVID‐19. Figure 1 excludes cases with missing stage. This is a descriptive figure designed to highlight absolute numbers only

The impact of the COVID‐19 pandemic on the volume of new diagnoses of breast, colorectal, lung, and prostate cancers is shown in Figure 1. Excluding cases with missing stage, 2120 (64.2%) were diagnosed pre‐COVID‐19, and 1184 (35.8%) were diagnosed in early‐COVID‐19, representing a proportional 44.2% decline in the numbers of new cases of breast, colorectal, lung, and prostate cancers, *p* < 0.0001.

Specifically, in breast cancer, 792 (65.1%) were diagnosed pre‐COVID‐19, and 424 (34.9%) were diagnosed early‐COVID‐19, representing a proportional 46.5% decline, *p* < 0.0001. In colorectal cancer, 265 (63.9%) were diagnosed pre‐COVID‐19, and 150 (36.1%) were diagnosed early‐COVID‐19, representing a proportional 43.5% decline, *p* < 0.0001. In lung cancer, 491 (59.4%) were diagnosed pre‐COVID‐19, and 336 (40.6%) were diagnosed early‐COVID‐19, representing a proportional 31.6% decline, *p* < 0.0001. Lastly, in prostate cancer, 572 (67.6%) were diagnosed pre‐COVID‐19, and 274 (32.4%) were diagnosed early‐COVID‐19, representing a proportional 52.1% decline, *p* < 0.0001.

### 3.3 Impact of the COVID‐19 pandemic on stage at presentation

Further, we evaluated the pandemic's effect on disease stage at presentation. Pre‐COVID‐19, 356 (16.8%) were diagnosed with metastatic disease, versus 241 (20.4%) also with metastatic disease in early‐COVID‐19, representing a proportional increase of 21.4% in the numbers of new cases of breast, colorectal, lung, and prostate cancers with metastatic disease, *p* = 0.01.

The unadjusted odd ratio of presenting with metastatic disease early‐COVID‐19 compared to pre‐COVID‐19 was 1.27 (95% CI 1.06–1.52). Adjustment for race, age, insurance type, site of disease, and study cohorts reduced the OR of metastatic presentation early‐COVID‐19 to 1.16 (0.95–1.42).

**FIGURE 2:**
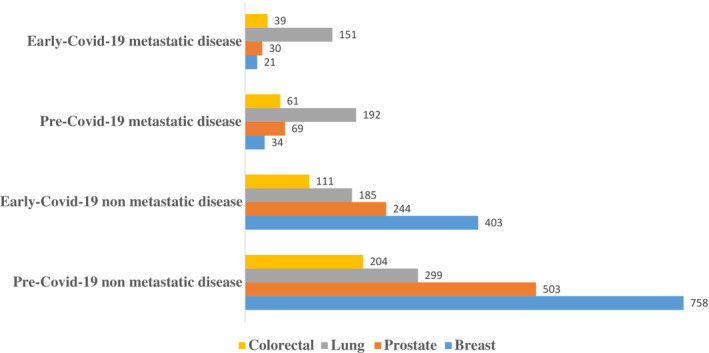
Impact of the COVID‐19 pandemic on stage at presentation according to disease site. Absolute numbers of new reported diagnoses of all breast, colorectal, lung, and prostate cancer pre‐COVID‐19 and early‐COVID‐19, and according to stage at disease presentation. Yellow bars represent colorectal cancers; gray bars represent lung cancer, orange bars represent prostate cancers, and blue bars represent breast cancers. Figure 2 excludes cases with missing stage. This is a descriptive figure designed to highlight absolute numbers only

There were no statistically significant increases in metastatic disease when the data was stratified by disease site (Table 2; Figure 2). Pre‐COVID‐19, 34 of 792 (4.3%) were diagnosed with metastatic breast cancer, versus 21 of 424 (5.0%) in early‐COVID‐19, representing a proportional increase of 16.2%, *p* = 0.60. Pre‐COVID‐19, 61 of 265 (23.0%) were diagnosed with metastatic colorectal cancer, versus 39 of 150 (26.0%) in early‐COVID‐19, representing a proportional increase of 13%, *p* = 0.50. Pre‐COVID‐19, 192 of 491 (39.1%) were diagnosed with metastatic lung cancer, versus 151 of 336 (44.9%) in early‐COVID‐19, representing a proportional increase of 14.8%, *p* = 0.09. Pre‐COVID‐19, 69 of 572 (12.1%) were diagnosed with metastatic prostate, versus 30 of 274 (11.0%) in early‐COVID‐19, representing a proportional 9.1% decline, *p* = 0.64.

**TABLE 2:** Impact of the COVID‐19 pandemic on stage at presentation according to disease site

	Pre‐Covid‐19, (%)	Early‐Covid‐19, (%)	Proportional difference^a^, %	*p* value
All cancers, non‐metastatic	1764 (83.2)	943 (79.7)	−4.2	0.01
Breast, non‐metastatic	758 (95.7)	403 (95.1)	−0.6	0.06
Colorectal, non‐metastatic	204 (77.0)	111 (74.0)	−3.9	0.50
Lung, non‐metastatic	299 (60.9)	185 (55.1)	−9.5	0.09
Prostate, non‐metastatic	503 (87.9)	244 (89.1)	1.4	0.64
All cancers, metastatic	356 (16.8)	241 (20.4)	21.4	0.01
Breast, metastatic	34 (4.3)	21 (5.0)	16.2	0.60
Colorectal metastatic	61 (23.0)	39 (26.0)	13.0	0.50
Lung, metastatic	192 (39.1)	151 (44.9)	14.8	0.09
Prostate, metastatic	69 (12.1)	30 (11.0)	−9.1	0.64
Whites, non‐metastatic	1469 (84.1)	800 (80.3)	−4.5	0.01
Blacks, non‐metastatic	231 (77.0)	126 (75.7)	−1.9	0.71
Whites, metastatic	278 (15.9)	196 (19.7)	23.9	0.01
Blacks, metastatic	69 (23.0)	41 (24.6)	7.0	0.71

^a^ Unadjusted difference derived by comparing early‐Covid‐19 with pre‐Covid‐19, negative values represent a decrease in the proportion of cases comparing early‐Covid‐19 with pre‐Covid‐19.

### 3.4 Impact of the COVID‐19 pandemic on stage at presentation according to race

Next, we evaluated the impact of the COVID‐19 pandemic on stage at presentation according to race. Pre‐COVID‐19, 278 of 1747 (15.9%) White patients were diagnosed with metastatic disease, versus 196 of 996 (19.7%) in early‐COVID‐19, representing a proportional 23.9% increase, *p* = 0.01. Pre‐COVID‐19, 69 of 300 (23.0%) Black patients were diagnosed with metastatic disease, versus 41 of 167 (24.6%) in early‐COVID‐19, representing a proportional 7.0% increase, *p* = 0.71. Table 3 shows unadjusted and adjusted changes in percentages of metastatic diagnoses according to race. Both unadjusted and adjusted difference‐in‐difference analyses showed no statistically significant differences in metastatic presentation comparing Black to White patients.

**TABLE 3:** Racial differences in percentages of metastatic presentation of breast, colorectal, lung, and prostate cancers comparing pre‐Covid‐19 to early‐Covid‐19

	Pre‐Covid‐19, (%)	Early‐Covid‐19, (%)	Difference^a^, % (95% CI)	Difference in differences^b^			
				Unadjusted		Adjusted^c^	
				%	*p* value	%	*p* value
White	278 (15.9)	196 (19.7)	3.8 (0.8, 6.8)	Ref		Ref	
Black	69 (23.0)	41 (24.6)	1.6 (−6.5, 9.6)	−2.2	0.61	0.0	1.00
Other	9 (12.3)	4 (19.1	6.7 (−11.7, 25.1)	3.0	0.76	1.3	0.86
Insurance: Private							
White	98 (12.7)	74 (16.3)	3.6 (−0.5, 7.8)	Ref		Ref	
Black	11 (10.4)	9 (13.6)	3.3 (−6.9, 13.4)	−0.4	0.95	0.4	0.96
Other	3 (7.9)	1 (12.5)	4.6 (−19.9, 29.1)	1.0	0.94	−2.0	0.93
Insurance: Medicare							
White	141 (17.0)	101 (22.0)	5.0 (0.4, 9.6)	Ref		Ref	
Black	37 (27.6)	23 (31.5)	3.9 (−9.2, 17.0)	−1.1	0.88	1.2	0.88
Other	3 (10.7)	2 (18.2)	7.5 (−18.0, 33.0)	2.5	0.85	2.6	0.87
Insurance: Medicaid							
White	18 (35.3)	12 (44.4)	9.2 (−13.7, 32.0)	Ref		Ref	
Black	14 (41.2)	8 (38.1)	−3.1 (−29.6, 23.5)	−12.2	0.49	−16.8	0.44
Other	1 (50.0)	1 (100.0)	50.0 (50.0, 50.0)	40.9		−3.6	0.97
Breast							
White	27 (4.1)	17 (4.8)	0.6 (−2.1, 3.3)	Ref		Ref	
Black	6 (5.6)	3 (5.3)	−0.3 (−7.6, 6.9)	−1.0	0.80	−0.2	0.98
Other	1 (2.9)	1 (8.3)	5.4 (−11.2, 22.0)	4.8	0.58	2.7	0.82
Colon							
White	51 (22.8)	28 (21.7)	−1.1 (−10.1, 7.9)	Ref		Ref	
Black	9 (26.5)	10 (50.0)	23.5 (−2.9, 50.0)	24.6	0.08	24.3	0.08
Other	1 (14.3)	1 (100.0)	85.7 (85.7, 85.7)	86.8		85.9	0.40
Lung							
White	153 (36.8)	129 (44.3)	7.6 (0.2, 14.9)	Ref		Ref	
Black	34 (53.1)	20 (51.3)	−1.8 (−21.7, 18.1)	−9.4	0.39	−5.3	0.62
Other	5 (45.5)	2 (33.3)	−12.1 (−60.0, 35.7)	−19.7	0.43	−27.9	0.28
Prostate							
White	47 (10.3)	22 (10.0)	−0.4 (−5.2, 4.5)	Ref		Ref	
Black	20 (21.1)	51 (15.7)	−5.4 (−18.3, 7.6)	−5.0	0.48	−5.9	0.53
Other	2 (11.5)	0 (0)	−11.5	−11.2	0.88	−9.2	0.89

^a^ Unadjusted difference derived by comparing early‐Covid‐19 with pre‐Covid‐19.

^b^ Derived by comparing Blacks with Whites.

^c^ Adjusted for age, insurance, disease site, and cohort.

## 4 DISCUSSION

The COVID‐19 pandemic resulted in widespread societal lockdowns leading to delays in seeking cancer screening and treatments.^22^, ^26^, ^28^, ^29^, ^30^, ^31^ A national United States (US) survey demonstrated that Black and Latinx adults with cancer self‐reported higher rates of cancer treatment delays and affordability of their cancer treatments.^32^ Black patients have been disproportionately affected by the COVID‐19 pandemic with higher rates of morbidity and mortality when compared to other racial groups.^33^, ^34^, ^35^, ^36^ As Black patients also have proportionally higher rates of late‐stage cancers compared with other races, we hypothesized that the COVID‐19 pandemic would further exacerbate racial disparities in cancer stage at presentation. We examined cancer registry data from three large US academic healthcare systems in order to quantify COVID‐19 related changes in new cancer volumes, stage at presentation, and racial differences in stages at presentation for cancers with established screening tests, including breast, colorectal, lung, and prostate cancers. To our knowledge, this report represents the first study to define race‐related changes in cancer presentation due to the COVID‐19 pandemic.

Several important findings emerged as a result of this study. We demonstrated a reduction in the volumes of new diagnoses reported for all four cancers, individually and combined. This is likely as a direct consequence of the abrupt cessation of cancer screening procedures at the height of the pandemic. Our results are consistent with another large cross‐sectional study that showed an almost equivalent proportional reduction of almost 50% for six cancers evaluated, including lung, colorectal, breast, pancreatic, esophageal, and gastric cancers.^37^ This reduction in volumes will correspondingly lead to later stages at presentation and subsequent poorer cancer outcomes, already being observed in other settings.^25^ Our data also demonstrated a COVID‐19 associated statistically significant decrease in the proportions of cancer cases presenting with non‐metastatic disease for the four cancers combined, with a corresponding rise in the proportions of cases presenting with metastatic disease. Prior estimates showed that through the end of 2020, accumulated deficits in new cancer cases and screening procedures persisted, with the exception of lung cancer screening that rebounded in the second half of 2020.^31^

Although Black patients bear a larger burden of COVID‐19 infections, hospitalizations, and deaths in the US, our difference in difference analyses suggested no racial differences in the burden of COVID‐19 related late‐stage cancer presentation after adjustments for age, insurance, disease site, and registry cohort. However, consistent with prior data, Black patients were still more likely to be diagnosed with metastatic disease than White patients. Following COVID‐19, the proportional reductions in non‐metastatic disease presentation and corresponding increases in metastatic disease presentation were not statistically significantly different in Black versus White patients. These data suggest that the COVID‐19 pandemic may have not exacerbated preexisting racial disparities in stage of presentation of cancers. A possible explanation for why we did not observe racial differences in late‐stage cancer presentation may be that since Black patients have been more susceptible to COVID‐19 related mortality, they may have died from COVID‐19 related illnesses rather than being diagnosed with metastatic cancers.^36^

The large multi‐institutional setting of the study merits comment and strengthens the impact of our study findings. Despite this strength, our study results must be interpreted in light of several limitations. The study was limited to only three academic institutions resulting in limited overall numbers and a limited proportion of Blacks; thus, results may not be generalizable to the entire US. The exclusion of data on Hispanic ethnicity also limit the interpretation of this study. Studies show that similar to Blacks, Hispanic or Latinx persons have also had increased rate ratios of cases, hospitalizations, and deaths compared to Whites.^36^ Due to the retrospective registry‐based nature of this study, we were not able to account for other confounding factors, and complete patient clinico‐pathological characteristics could not be adequately analyzed. Only cases with known race were retrieved from the databases. Although unlikely, it is possible that those with missing race from all three registries had different characteristics leading to selection bias. While we acknowledge that the first covid‐19 wave occurred at different times in different locations, essentially all of the US was on lockdown during the early‐COVID‐19 period we selected. The limited pre‐COVID‐19 and early‐COVID‐19 durations (4 months each) we evaluated may be insufficient to determine the full impact of the COVID‐19 pandemic on volume and shifts in cancer stages at presentation. As lockdowns continued, it is possible that the incidence of late‐stage diagnoses increased. The limited duration we evaluated also did not allow us to study any differences in long‐term outcomes, if any subsequent rebound in volumes of new diagnoses occurred with time, or if reversals to pre‐COVID‐19 stages at presentation ensued.

In conclusion, the COVID‐19 pandemic has resulted in major disruptions and delays across the cancer care continuum in the US and globally. Although the impact of the COVID‐19 pandemic predictably led to a reduction in the volume of new cancer diagnoses and a stage migration to more advanced disease presentation, we did not find any racial differences in the observed proportion of cancers presenting at a metastatic stage due to the COVID‐19 pandemic. Additional research in other cancers is needed to understand the full impact of the pandemic on cancer care trajectories and disparities, and further research is needed to define future mitigation plans to avoid negative long‐term cancer outcomes from future public health crises.

## AUTHOR CONTRIBUTIONS

**Jennifer Berrian:** Data curation (equal); investigation (equal); project administration (equal); writing – original draft (equal); writing – review and editing (equal). **Ying Liu:** Data curation (equal); formal analysis (equal); investigation (equal); methodology (equal); software (equal); writing – original draft (equal); writing – review and editing (equal). **Nkiruka Ezenwajiaku:** Data curation (equal); resources (equal); writing – original draft (equal); writing – review and editing (equal). **Alvaro Moreno‐Aspitia:** Data curation (equal); resources (equal); writing – original draft (equal); writing – review and editing (equal). **Sara J Holton:** Data curation (equal); resources (equal); writing – original draft (equal); writing – review and editing (equal). **Adetunji T. Toriola:** Writing – original draft (equal); writing – review and editing (equal). **Graham A. Colditz:** Writing – original draft (equal); writing – review and editing (equal). **Ashley J. Housten:** Writing – original draft (equal); writing – review and editing (equal). **Lannis Hall:** Writing – original draft (equal); writing – review and editing (equal). **Mark Fiala:** Writing – original draft (equal); writing – review and editing (equal). **Foluso O. Ademuyiwa:** Conceptualization (lead); data curation (lead); formal analysis (supporting); investigation (lead); methodology (lead); project administration (lead); resources (lead); supervision (lead); writing – original draft (lead); writing – review and editing (lead).

## FUNDING INFORMATION

None.

## CONFLICT OF INTEREST

None.

## Data Availability

Data sharing is not applicable to this article as no new data were created or analyzed in this study.
